# The TβR II-targeted aptamer S58 prevents fibrosis after glaucoma filtration surgery

**DOI:** 10.18632/aging.102997

**Published:** 2020-05-23

**Authors:** Xueru Li, Yu Leng, Xiangji Li, Yawei Wang, Peng Luo, Chi Zhang, Ziwen Wang, Xiaofeng Yue, Chongxing Shen, Long Chen, Zujuan Liu, Chunmeng Shi, Lin Xie

**Affiliations:** 1 Department of Ophthalmology, The Third Affiliated Hospital of Chongqing Medical University (Gener Hospital), Chongqing 401120, China; 2 Institute of Rocket Force Medicine, State Key Laboratory of Trauma, Burns and Combined Injury, Third Military Medical University, Chongqing 400038, China

**Keywords:** glaucoma filtration surgery (GFS), aptamer, S58, fibrosis, Nrf2/PI3k/Akt

## Abstract

Glaucoma filtration surgery (GFS) is an effective clinical treatment for glaucoma when intraocular pressure (IOP) control is poor. However, the occurrence of conjunctival scarring at the surgical site is the main reason for failure of the surgery. In a previous study, we isolated and developed S58, a novel nucleic acid aptamer targeting TβR II, by systematic evolution of ligands by exponential enrichment (SELEX). Here, we show how S58 sterically inhibits the TβR II interaction with TGF-β. The effects of topical S58 treatment were studied in a rabbit model of GFS. At 6 postoperative weeks, S58 reduced fibrosis and prolonged bleb survival in rabbits after GFS. Further in vitro tests showed that the levels of fibrosis in S58 treated-Human Conjunctival Fibroblasts (HConFs) were decreased and that antioxidant defense was increased. In addition, the loss of nuclear factor erythroid 2-related factor 2 (Nrf2) or the inhibition of phosphoinositide 3-kinase/protein kinase B (PI3K/Akt) reversed the anti-fibrotic effects of S58. The present work suggests that S58 could effectively improve GFS surgical outcomes by activating the intracellular antioxidant defense PI3K/Akt/Nrf2 signaling pathway.

## INTRODUCTION

Glaucoma is a common disease of the elderly and one of the most common causes of irreversible blindness worldwide, affecting patients’ visual function and quality of life [1–3]. Glaucoma filtration surgery (GFS) is the most effective treatment for drug-refractory glaucoma [4–6]. However, surgery is not always successful due to conjunctival scarring at the surgical site. During the operation, the destruction of the vasculature of the conjunctival tissues and the stimulating effects of exudates and hormones on the conjunctival fibroblasts are important causes for postoperative filtration bleb fibrosis [7–10]. Several clinical anti-fibrosis drugs, such as steroids, 5-Fluorouracil (5-FU), and mitomycin C (MMC) are widely used in the clinic, but side effects such as postoperative low intraocular pressure (IOP), endophthalmitis, and anterior chamber leakage limit their applications [11]. It is urgent to develop safe and more effective strategies to improve wound healing and inhibit scar formation after GFS.

Over the past few years, aptamers have attracted widespread attention [12, 13]. Aptamers, single-stranded DNA or RNA oligonucleotides screened by SELEX, are similar to conventional antibodies that specifically bind to a target protein [14]. The function of the aptamer-target binding depends on structural compatibility, electrostatic and van der Waals interactions, stacking of aromatic rings, hydrogen bonding, or some combination thereof [15]. In addition, aptamers are chemically and biologically unique, non-toxic, and easy to synthesize [16]. Pegaptanib (Macugen), the first FDA-approved RNA aptamer, targets the VEGF_165_ isoform for the treatment of age-related macular degeneration (ARMD) [17–19]. Recently, a novel anti-angiogenic and anti-scarring dual action of an anti-fibroblast growth factor 2 aptamer was established in animal models of retinal disease [20]. Moreover, several cell-specific aptamer-binding targets have also been identified and used in diagnosis and therapy, such as PCBP2 for liver fibrosis [21], RAGE-aptamer for diabetic nephropathy [22], and RB011 for bleomycin-induced pulmonary fibrosis [23].

As features of scar formation after GFS, the levels of activated TGF-β and TGF-β receptor II (TβR II) are significantly elevated at the wound site [24–26], which is critically important to the process of filtration bleb scar information. In a previous study, we employed SELEX to isolate and develop an ssDNA aptamer, termed S58, which showed high binding affinity for TβR II [27]. We know that risk factors for scar-related failure include previous surgical procedures destroying the conjunctival structure, a longer-term topical conjunctival medication, and conjunctival inflammation. Thus, modulating conjunctival wound healing has the potential to improve outcomes after GFS. Recent studies have aroused concern about the role of reactive oxygen species (ROS) and oxidant-antioxidant balance in modulating TGF-βs-induced fibrosis and indicate that increased oxidative stress is critical for the development of fibrotic diseases [10, 27–31]. Some researchers have also shown that the intraocular oxidant-antioxidant balance is altered after GFS and damage/oxidative stress to the trabecular meshwork increases risk of glaucoma [28, 32]. Therapeutics targeting both TGF-β-induced and ROS-dependent cellular oxidative stress signaling may provide a novel approach for the treatment of fibrotic disorders. Here, we ask whether S58 promotes the antioxidant capability of damaged cells and could improve the outcomes of GFS.

## RESULTS

### S58 reduces fibrosis by targeting TβR II

**Figure 1:**
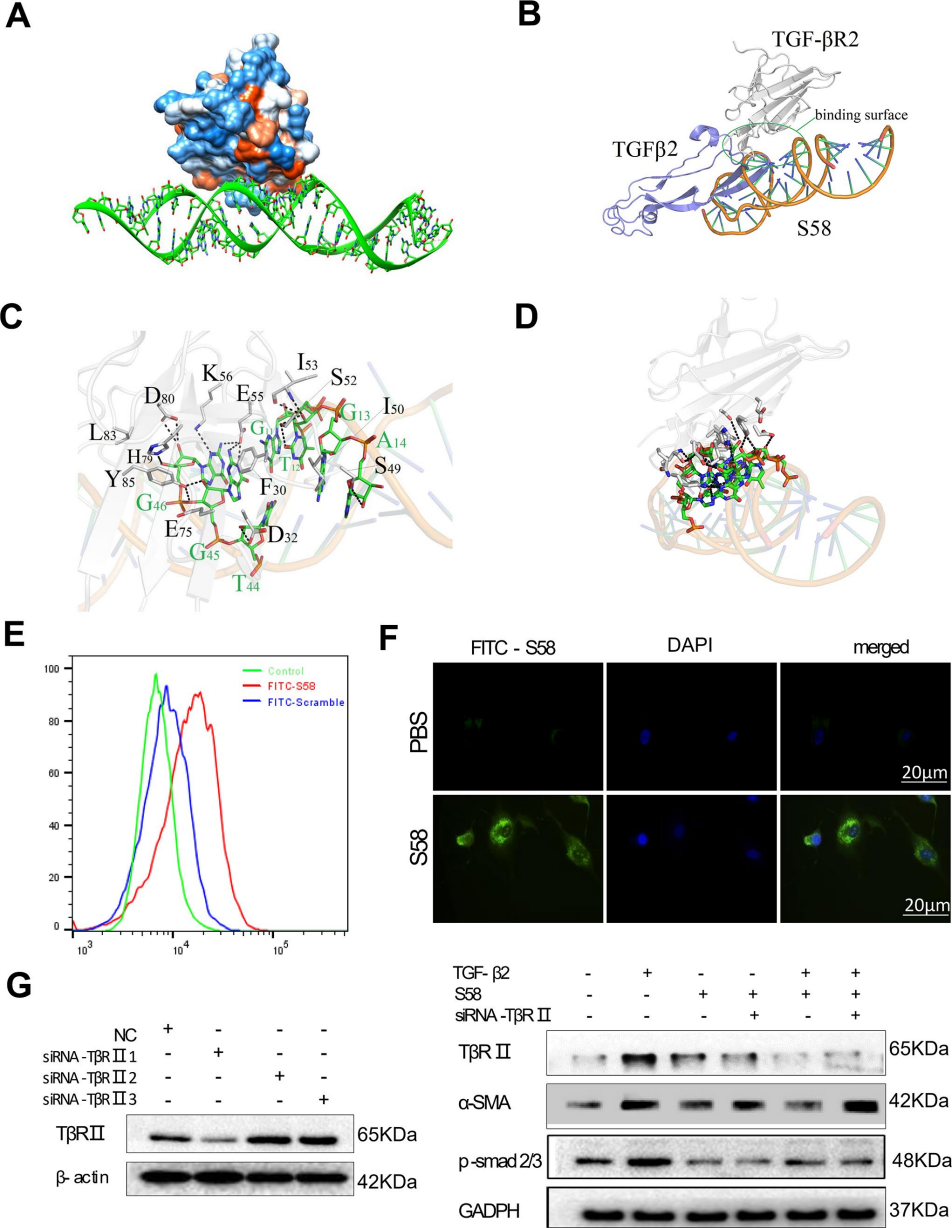
**Determination of the interaction between TβR II and S58.** (**A**) Hydrophobic surface properties between TβR II and S58. (**B**) Binding sequence between TβR II and S58. (**C**) The TβR II and S58 interaction regions. (**D**) Conformation superposition of crystalline TβR II, TGF-β2 and S58. (**E**) Representative images showing immunofluorescence staining for S58 targets TβR II receptor (nuclei stained blue, FITC-S58 stained green). (**F**) HConFs were pretreated by TGF-β2 with or without in the presence of S58 and were transfected with or without siRNA-TβR II for 72. (**G**) Protein levels of α-SMA, TβR II and p-smad2/3 were determined. GAPDH was used as loading control.

The lowest energy and best phase structure were selected from 1000 blind docking results for subsequent analysis (Supplementary Figure 1B). The results obtained prompted us to explore the dynamic behavior of the S58-TβR II complex. Supplementary Figure 1C shows the complex structure with the lowest combination score in the exact docking process. Polar interactions primarily involved stabilizing the complex by non-bonded electrostatic interactions (Figure 1A). The complex structure between TβR II and TGF-β1/2 was downloaded from the RCSB PDB database. The conformational superposition of the S58, TβR II, and TGF-β1/2 crystal structures indicated that S58 had a significant spatial collision with the native TGF-β1/2 (Figure 1B, 1D and Supplementary Figure 1D, 1E). Amino acid residues in the target protein TβR II interacting with the selected S58 conformation were counted to predict the protein function point. There were 12 amino acids of the protein TβR II (Y85, E75, L83, D80, K56, E55, I53, S52, I50, S49, D32, F30), which bound to the interface region and formed an important interaction with S58 (G11, T12, G13, A14, G46, G45, T44). Multiple conformation superposition results showed that the binding sites between S58 and TβR II were relatively concentrated. TβR II formed a strong interaction with S58, including hydrophobic and polar interactions and hydrophobic/hydrophilic surface complementarity; 12 amino acids were identified as driving interactions and stabilize the TβR II-S58 complex (Figure 1C, 1D). Therefore, S58 blocked the binding between TGF-βs and TβR II.

A strong fluorescence shift was observed in FITC-S58 (200nM, 1h on ice)-incubated HConFs versus FITC-scramble incubation (Figure 1E). Immunofluorescence results revealed that HConFs had stronger green fluorescence after S58 incubation than PBS-treated cells, indicating that S58 could bind to receptor proteins on the HConFs surface (Figure 1F). HConFs pretreated with S58 (20nM) followed by TGF-β2 (4ng/ml) treatment were transfected with or without siRNA-TβR II for 72h. Knockdown of TβR II abolished the binding of S58 (Figure 1G). Furthermore, the protein levels of α-SMA, TβR II and p-samd2/3 were significantly elevated with S58 treatment followed by siRNA-TβR II precondition. Together these results indicate that targeting TβR II was the key to S58 inhibiting TGF-β2-induced fibrosis.

### S58 prolongs the survival of filtering blebs in rabbits after GFS

**Figure 2:**
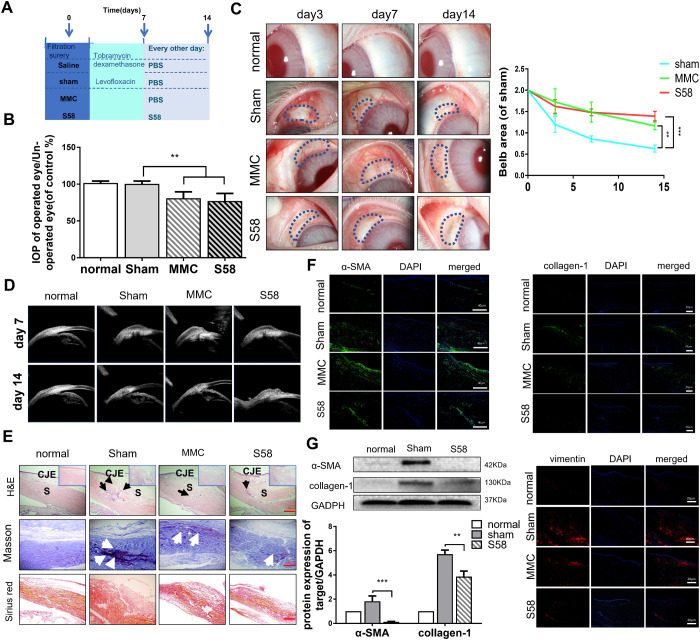
**S58 treatment prolongs the survival of filtering blebs after GFS in rabbits.** (**A**) Animal model establishment and postoperative model of administration. (**B**) IOPs of the operated and non-operated eyes were measured. The numbers indicated the mean IOP percentage of the control group (n=5). (**C**) Representative stereo-microscopic images showing bleb characteristics in different groups of rabbits’ eyes (the area of blebs is outlined by dotted blue lines) at day 3, 7, and 14 after GFS, and quantization of bleb area. (**D**) UBM imaging of the rabbits’ conjunctiva confirmed bleb survival. (**E**) Representative images of H&E (shown by black arrow), Masson’s trichrome staining (as white arrow showed) and Sirius Red staining (n=5). (**F**) Representative images showing immunohistochemical staining for α-SMA, vimentin, and collagen-1 in rabbits’ ocular tissue sections, collected at day 14 after GFS. (Nuclei = blue, vimentin = red, and α-SMA/ collagen-1= green). (**G**) Fibrotic protein levels of α-SMA/collagen-1 was examined by western blotting. GAPDH was used as loading control. n = 3. Data indicate the mean ± SD. *p < 0.05, **p < 0.001.

Our animal treatment program is shown in Figure 2A. Topical application of S58 (20nM/drop) 4 times daily significantly improved the outcomes in rabbits after GFS (Supplementary Figure 2). Analysis of mean IOP in the surgical eyes showed no significant differences between the MMC and S58 groups on postoperative day 14, but the IOP values of MMC group were higher than the sham and S58 groups after 6 postoperative weeks (Figure 2B and Supplementary Figure 3C). Slit lamp examination showed the typical appearance of the blebs at the 1 week, 2 weeks, 3 weeks, 4 weeks, 5 weeks, and 6weeks postoperative endpoints. Representative images showed that the S58 and MMC groups exhibit significantly prolonged bleb survival compared to the sham group. There are slightly bulged, thinner blebs and scleral flap were clearly visible in the MMC-treated and S58-treated groups. Conversely, the blebs were small, thick, flat, and vascularized in the sham group. The total area of functional filtration bleb in the S58 group was significantly larger than in the MMC group, except at postoperative day 7 (Figure 2C and Supplementary Figure 3B). The ultrasound biomicroscopy (UBM) was used to further observe the cystic structure of the conjunctival filtration bleb. A cystic cavity was seen under the conjunctiva of the operation area. During postoperative week 6, the conjunctival tissues were thickened in the sham group compared to S58 group, and no obvious cavities were observed under the sham group. A limited cystic cavity was still visible in the MMC group and the 58 group (Figure 2D and Supplementary Figure 3A).

**Figure 3:**
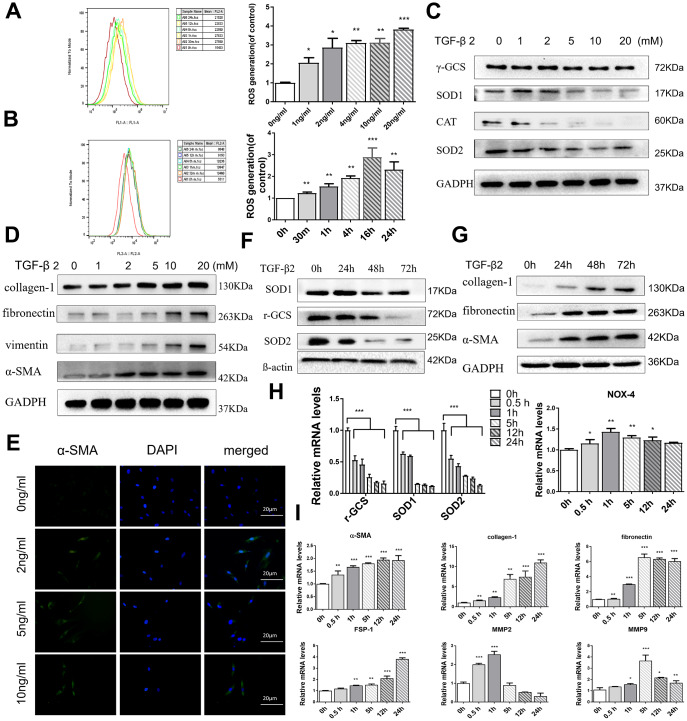
**TGF-β2 increases oxidative stress and induces fibrosis in HConFs.** (**A**) Mitochondrial superoxide variation, and (**B**) intracellular ROS variation at indicated TGF-β2 treatment. (**C**) Intracellular antioxidant protein SOD1/2, CAT, γ-GCS, and (**D**) fibrotic protein α-SMA, fibronectin, vimentin, and collagen-1 expression variation at different TGF-β2 concentration precondition for 24h. (**E**) Representative images showing immunofluorescence staining for α-SMA generated after preconditioning with different TGF-β2 concentrations for 24h (Nuclei=blue, α-SMA=green). (**F**) Levels of antioxidant proteins SOD1/2 and γ-GCS levels and (**G**) fibrotic proteins α-SMA, fibronectin, and collagen-1. (**H**) Levels of oxidative stress-related genes SOD1/2, γ-GCS and NOX4 and (**I**) fibrosis-related genes α-SMA, fibronectin, and collagen-1, FSP-1, MMP-2, MMP-9. n=3. All data indicate the mean ± SD. *p < 0.05, **p < 0.01, ***p < 0.001.

Bleb sections were stained with hematoxylin and eosin (H&E) (Figure 2E, black arrow), Masson’s trichrome staining (Figure 2E, white arrow), and Sirius red staining. The ratios of myofibroblasts to total cells and the formation of collagen tissue in the MMC-treated and S58-treated groups were significantly attenuated compared to the sham group. Sham group exhibited severe scarring at surgical sites (Figure 2E), including more inflammatory cells and proliferating cells in the scleral gap and bleb area. In addition, Masson’s trichrome staining and Sirius red staining of sham-treated eyes exhibited nearly severe scarring at surgical sites, including evidence of more collagen deposition in the scleral gap and bleb area compared to MMC treatment and S58 treatment. Immunofluorescence staining showed higher expression of α-SMA, vimentin, and collagen-1 in sham group than MMC and S58 group (Figure 2F). S58 significantly decreased the fibrotic protein levels of α-SMA and collagen-1 relative to the sham group, suggesting fewer myofibroblasts at surgery sites (Figure 2G). The above data suggest that S58 reduces fibrosis in rabbits after GFS.

### TGF-β2 increases oxidative stress and fibrosis of HConFs

Previous reports have indicated that exposure to TGF-βs could increase intracellular ROS and sustained cellular stress response, increasing the occurrence of EMT and ultimately inducing tissue fibrosis [33, 34]. By flow cytometry analysis, the MitoSOX red and 2’,7’-dichlorofluorescin diacetate (H_2_DCF-DA) assay revealed that HConFs, with different TGF-β2 treatment times and concentrations, provoked a sustaining release of mitochondrial and cytosolic superoxide that remained higher than the untreated group, indicating that TGF-β2-induced HConFs were in a state of sustained high stress (Figure 3A, 3B). Moreover, TGF-β2 precondition significantly decreased levels of antioxidants involved in ROS scavenging, SOD1/2 (Superoxide dismutase) and γ-GCS (γ-glutamyl cysteine synthetase), in a time and dose-dependent manner (Figure 3C, 3F). We saw a positive correlation between the expression of α-SMA and the concentration of TGF-β2 (Figure 3E). In contrast, TGF-β2 treatment of HConFs significantly increased the protein and gene expression of fibrosis associated markers in a time and dose-dependent manner. However, MMP-2 and MMP-9 gene expression peaked at 1h-5h and then decreased (Figure 3D, 3G, 3I). Antioxidation-related gene expression continuously decreased over time in TGF-β2-induced HConFs (Figure 3H).

### ROS plays an important role in TGF-β2-induced HConFs fibrosis

**Figure 4:**
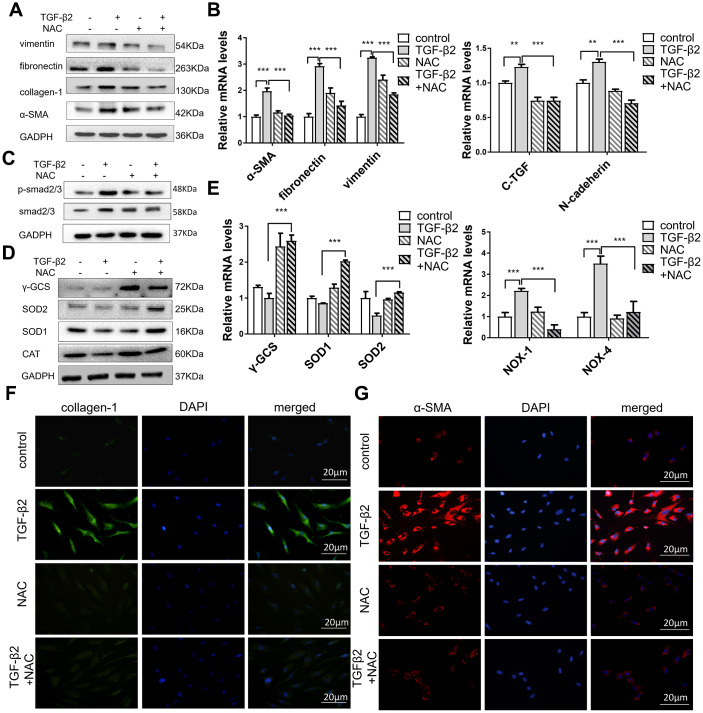
**NAC alleviates TGF-β2-induced fibrosis in HConFs by improving cell antioxidant defense.** HConFs were pretreated by TGF-β2 (4 ng/ml) with or without NAC (10 mM) for 24 h. (**A**) Protein levels of vimentin, α-SMA, fibronectin, and collagen-1. (**B**) mRNA levels of fibronectin, vimentin, C-TGF, N-cadherin and α-SMA determined by quantitative real-time PCR. (**C**) NAC effect on p-smad2/3 protein levels in TGF-β2-induced HConFs. (**D**) Relative antioxidant protein levels of γ-GCS, SOD1/2 and CAT determined using western blotting. (**E**) Relative antioxidant gene levels of γ-GCS, SOD1/2, NOX-1, and NOX-4 at specified times. (**F**) Representative images showing immunofluorescence staining for collagen-1 and (**G**) α-SMA in TGF-β2-induced HConFs at 24h (Nuclei = blue, α-SMA/collagen-1 = green/red). Data are mean ± S.D. n = 3. *p < 0.05, **p < 0.01, ***p < 0.001.

Previous reports have shown that oxidant-antioxidant regulation imbalance is key to oxidative stress in ROS, causing fibrotic diseases [34, 35], and the main cause of TGF-βs-induced oxidative stress damage is the accumulation of ROS. Acetylcysteine (N-acetyl-l-cysteine) (NAC) inhibits the production of ROS of HConFs in a high-glucose environment [36]. We observed that NAC significantly reduced α-SMA, vimentin, fibronectin, and collagen-1 protein levels in TGF-β2-induced HConFs (Figure 4A) and expression of fibronectin, vimentin, C-TGF (a potent fibrotic cytokine), N-cadherin, and α-SMA genes (Figure 4B). NAC also inhibited p-smad expression (Figure 4C). We further observed that NAC treatment reversed TGF-β2-induced changes in γ-GCS, SOD1/2 and CAT protein and gene levels in HConFs (Figure 4D, 4E). NAC may inhibit TGF-β2-induced stress by increasing the gene and protein levels of SOD1/2 and γ-GCS. Moreover, the results of NOX-1 and NOX-4 confirmed the decrease of ROS in TGF-β2-induced HConFs. Immunofluorescence staining of α-SMA and collagen-1 revealed that NAC significantly alleviated the levels of TGF-β2-induced fibrosis (Figure 4F, 4G). We could conclude that NAC blockade of ROS production inhibited TGF-β2-induced HConFs fibrosis. The lower level of ROS is closely associated with a higher success rate of surgery.

### S58 promotes antioxidant defense of TGF-β2-induced HConFs

**Figure 5:**
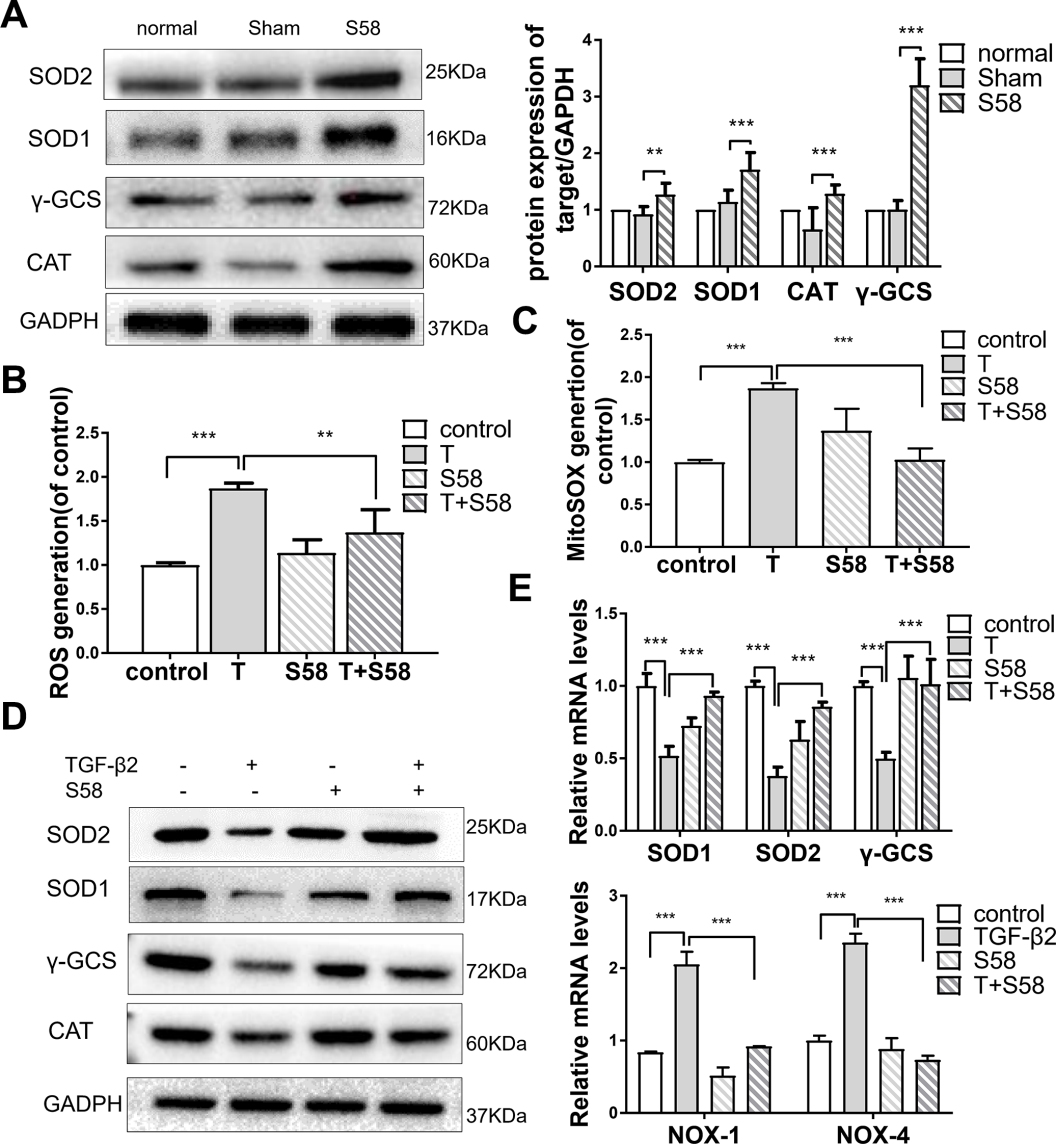
**S58 promotes antioxidant defense of TGF-β2-induced HConFs.** (**A**) Analysis of antioxidant capacity of cells at day 14 after GFS. (**B**) Intracellular ROS variation, and (**C**) mitochondrial superoxide variation were examined by flow cytometry. (**D**) Antioxidant protein SOD1/2, γ-GCS and CAT levels analyzed by western blotting in TGF-β2-treated HConFs in the presence or absence of S58 (20 nM). (**E**) Relative antioxidant gene levels in HConFs preconditioned with TGF-β2 in the presence or absence of S58 (20 nM) for 12h. All data indicate the mean ± SD, n=3. *p < 0.05, **p < 0.01, ***p < 0.001.

Increased oxidative stress is crucial to the development of human fibrosis [37]. TGF-βs, the essential cytokine in fibrosis development, increase ROS production in fibroblasts [38, 39]. On postoperative day 14*,* in vivo study showed that the levels of the antioxidant proteins SOD1/2, CAT, and γ-GCS in the S58 treatment group were higher than in controls (Figure 5A). S58 obviously attenuated mitochondrial and cytosolic superoxide accumulation in HConFs (Figure 5B, 5C). The levels of different antioxidants involved in ROS scavenging and SOD1/2, γ-GCS, and CAT levels were significantly elevated in TGF-β2-induced HConFs after S58 precondition (Figure 4D, 4E), suggesting that S58 attenuation of ROS damage is cytoprotective.

### S58 reduces TGF-β2-induced HConFs fibrosis

**Figure 6:**
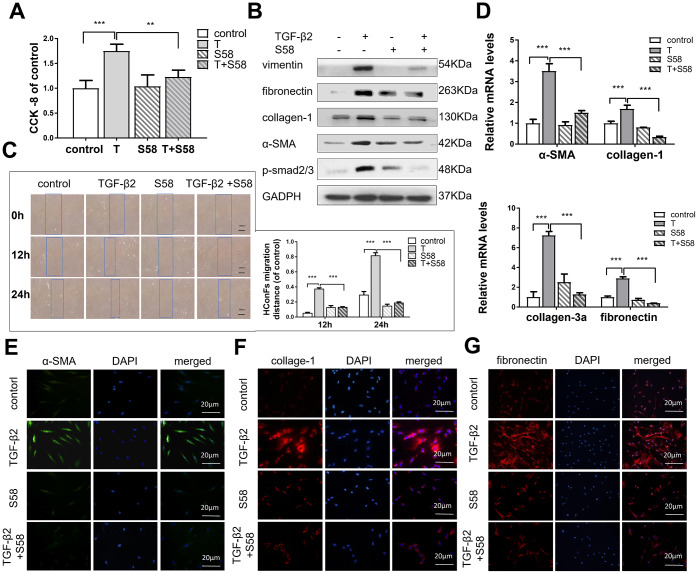
**S58 reduces TGF-β2-induced HConFs fibrosis.** (**A**) Effect of TGF-β2 and S58 on HConFs viability (**B**) Western blot of fibrosis-related proteins. (**C**) Representative images and quantification of cell motility of TGF-β2-treated HConFs with or without the presence of S58 at specified times (Dotted blue lines: edges of the migrated cells). (**D**) mRNA levels of fibronectin, collagen-1, α-SMA and collagen-3a. (**E**–**G**) Levels of α-SMA, fibronectin, and collagen-1 were analyzed by immunofluorescence staining after 24h treatment (Nuclei = blue, α-SMA = green, fibronectin/collagen-1 = red). Data indicate the mean ± SD. n=3. *p < 0.05, **p < 0.01, ***p < 0.001.

Conjunctival fibrosis plays an equally important role in scarring after GFS [6, 7]. TGF-β2 significantly increased HConFs viability, while S58 reversed this increase dramatically (Figure 6A). Furthermore, S58 obviously decreased TGF-β2-induced HConFs fibrosis. Expression of the fibrosis-related proteins vimentin, fibronectin, collagen-1, α-SMA, and p-smad2/3 were significantly reduced in TGF-β2-treated HConFs with the presence of S58 (Figure 6B). Time-lapse imaging showed that S58 significantly alleviated HConFs motility activities (Figure 6C). S58 treatment reduced expression of fibrotic genes in HConFs (Figure 6D). S58 reduced the immunofluorescence intensity of α-SMA, fibronectin, and collagen-1 in TGF-β2-treated cells (Figure 6E–6G). We conclude that S58 inhibited TGF-β2-induced fibrosis of HConFs by inhibiting cell activity, migration ability, and expression of fibrosis-related proteins and genes.

### S58 reverses TGF-β2-induced HConFs fibrosis via activating the PI3K/Akt/Nrf2 signaling pathway

**Figure 7:**
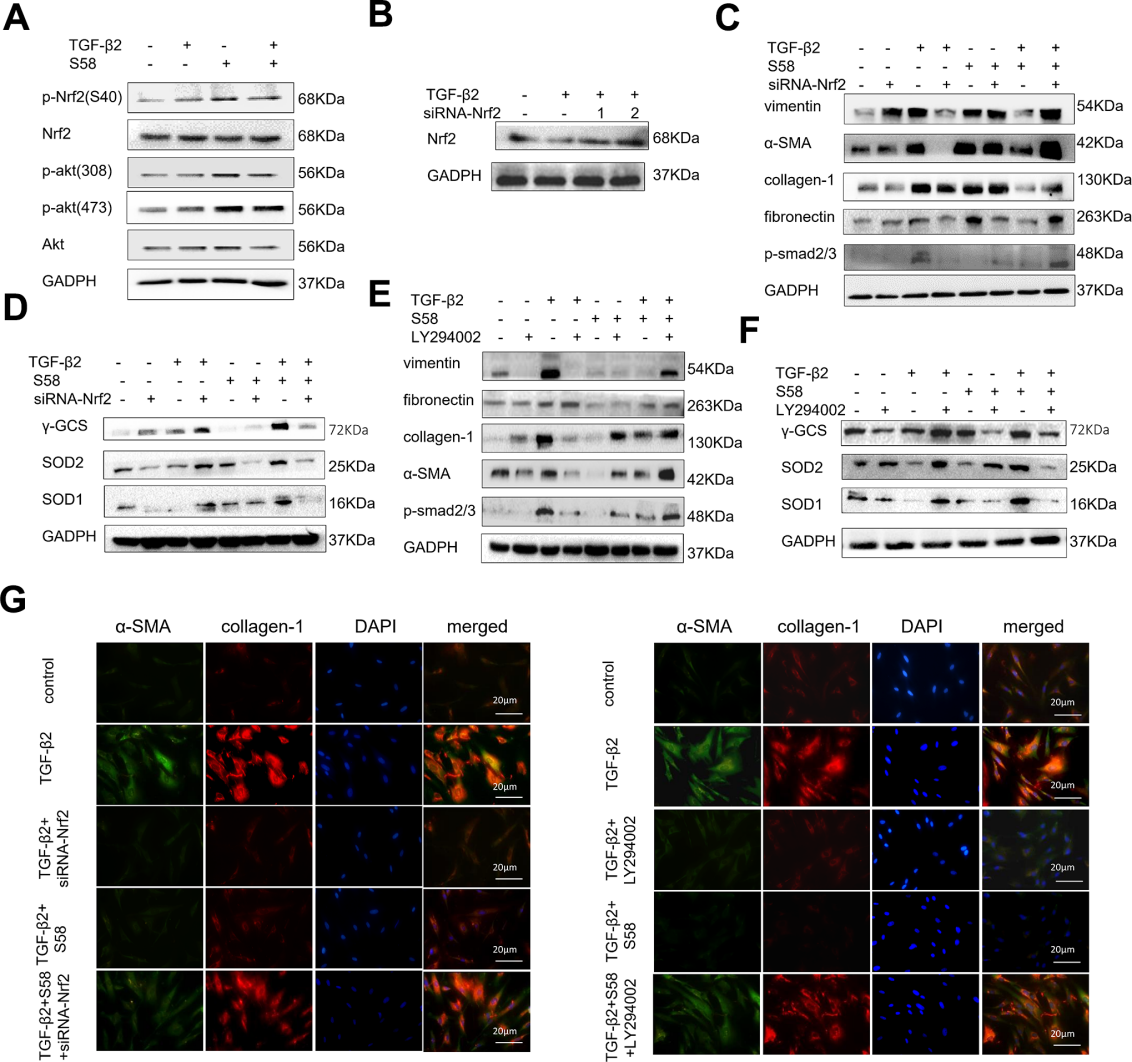
**S58 promotes antioxidant defense against TGF-β2-induced fibrosis in HConFs via the activation of PI3K/Akt/Nrf2 signaling pathway.** (**A**) p-Nrf2(S40), Nrf2, Akt, p-Akt (308) and p-Akt (473) levels in cell lysates from TGF-β2-pretreated HConFs for 24h. (**B**) Screening for specific siRNAs to knock down Nrf2 protein. (**C**, **E**) Relative levels of fibrotic proteins and (**D**, **F**) antioxidant defense proteins in whole cell lysates from S58-treated HConFs with/without siRNA-Nrf2 transfection(or LY294002 (40×10^-6^ m) after 72h. (**G**) Fibrosis levels were analyzed by co-staining of α-SMA and collagen-1 immunofluorescence.

It has been reported that redox homeostasis is maintained by the activation of Nrf2, and its downstream transcriptional targets [40]. Nrf2 activation increases the expression of multiple transcription factors associated with antioxidant, anti-inflammatory, and other cytoprotective pathways by binding to the antioxidant response element [41]. We found that S58 significantly increased phosphorylation of Akt and promoted phosphorylation of Nrf2 expression (Figure 7A). Furthermore, LY294002 (a PI3K/Akt inhibitor) and siRNA-Nrf2 (Figure 7B) were applied to explore the possible involvement of the PI3K/Akt/Nrf2 signaling pathway in adjusting the oxidative stress of HConFs. Precondition with siRNA-Nrf2 / LY294002 significantly decreased the anti-fibrosis capacity of S58 (Figure 7C, 7E) and decreased expression of intracellular antioxidants (Figure 7D, 7F). Immunofluorescence staining confirmed the important role of activating PI3K/Akt/Nrf2 signaling pathway in S58 anti-fibrosis (Figure 7G). Together, the above data indicate that S58, specific targeting TβR II, inhibits fibrosis and activates PI3K/Akt/Nrf2 signaling pathway in HConFs.

## DISCUSSION

GFS is the main clinical treatment for refractory glaucoma [4]. However, patients with controlled IOP may still suffer from filtering surgery failure due to excessive scar formation of the overlying conjunctiva [8]. Thus, modulation of conjunctival wound healing may improve outcomes after GFS. Antimetabolites, especially MMC and 5-FU, are usually applied intraoperatively and/or postoperatively as an adjunct to inhibit subconjunctival fibrosis following GFS [42], but due to their non-specificity, these antimetabolites sometimes cause severe side effects [11].

In recent years, aptamers have been applied toward cancer diagnosis, bio-marker discovery, targeted therapy, and drug delivery [20, 43]. Characterized by their flexible chemical modification, aptamers are conjugated to other chemicals, such as siRNA, nanoparticles, chemotherapeutic agents, and solid phase surfaces for therapeutic and diagnostic applications [14]. With the advent of individualized therapy, aptamer targeted therapy has attracted widespread attention. TGF-βs are well-known cytokines that promote scar formation, and activated TGF-β2 and TβR II are elevated at the wound site [28, 29]. Our previous studies revealed that the aptamer S58 inhibited TGF-β2-induced transdifferentiation of human Tenon’s capsule fibroblasts (HTFs) by increasing steric hindrance of TβR II [44].

Oxidant-antioxidant imbalance plays a key role in human tissue fibrosis [45, 46]. Redox homeostasis is primarily maintained by PI3K/Akt-dependent activation of Nrf2 and its downstream transcriptional targets [47]. Nrf2 controls ROS and other cellular stresses, ultimately improves the cell antioxidant defense to regulate cell growth, self-renewal, differentiation, and proliferation for TGF-β-induced EMT [48, 49].

**Figure 8:**
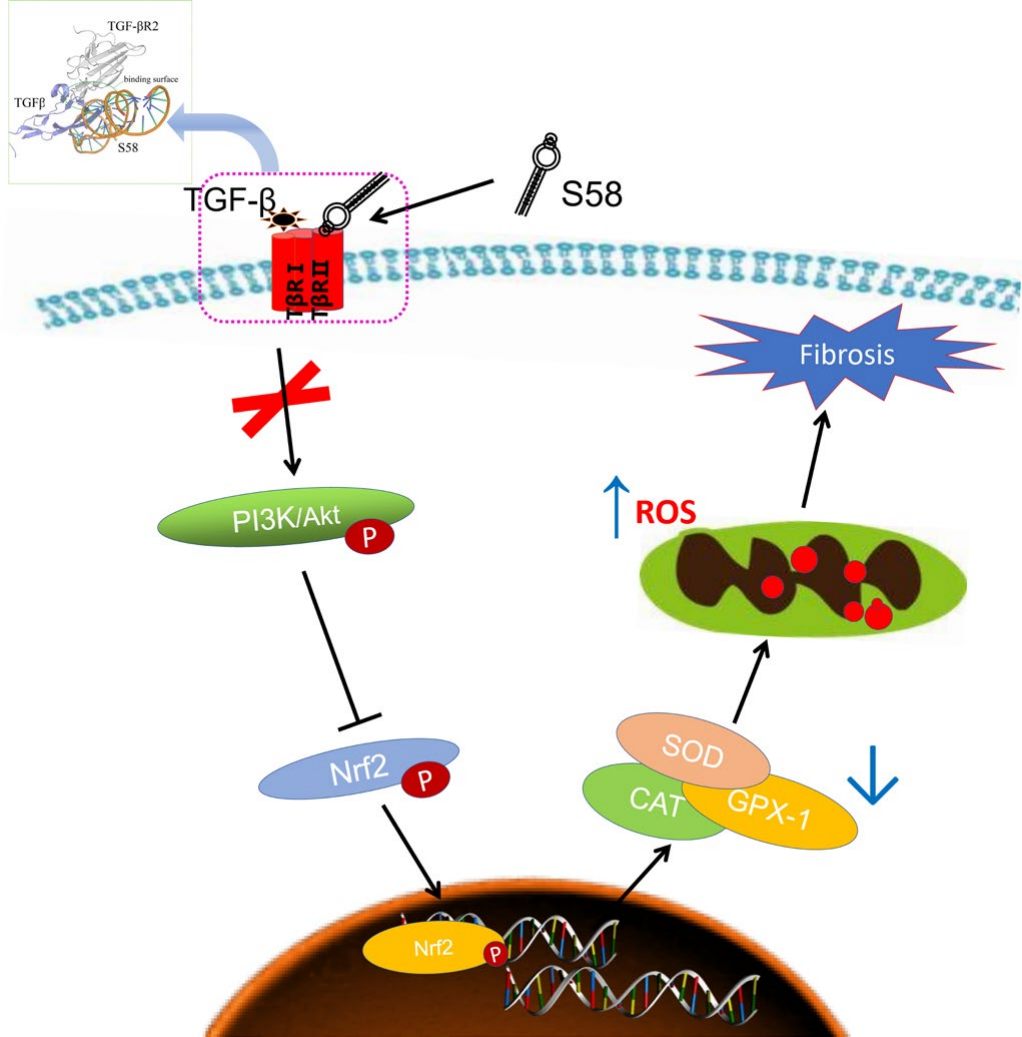
**Schematic diagram showing the mechanisms by which S58 promotes antioxidant defense of HConFs against fibrosis.** S58 competitively binds to TβR II at the expense of TGF-β2 obviously accelerates the removal of ROS by activating the PI3K/Akt/Nrf2 signaling pathway and improves repair potential.

In our present study, we investigated the potential anti-fibrotic effects of S58 in rabbits after GFS and in TGF-β2-induced HConFs. Our results are consistent with previous studies that demonstrated that TGF-β2 induced cell proliferation and myofibroblast differentiation and migration [27]. A previous article reported that the anti-fibrotic effect is closely related to the endogenous antioxidant defense [48, 49]. Because TGF-βs significantly increased cellular oxidative stress, inhibiting ROS production with NAC could effectively reduce fibrosis [50]. We observed fewer myofibroblasts, less collagen deposition, and more antioxidant protein expression at the surgical area in the S58 group compared with sham and MMC groups. Further, S58 significantly reduced TGF-β2-induced fibrosis and enhanced antioxidant capability in HConFs. Consistent with these findings [29], ROS plays a critical role for TGF-β-induced smad phosphorylation and myofibroblastic differentiation. In this study, the loss of Nrf2 or inhibition of PI3K/Akt significantly inhibited the anti-fibrosis capacity of S58. The present work suggests that S58 potentially reduced scar formation of filtering bleb by improving the antioxidant capacity of damaged cells (Figure 8).

## MATERIALS AND METHODS

### Molecular docking

Documentation of protein structure information (https://www.uniprot.org/uniprot/P37173.fasta, https://www.uniprot.org/uniprot/P01137.fasta, http://www.prof.org/uniprot/P10600.fasta). The secondary structure of S58 was predicted according to its nucleotide sequence (Supplemental Figure 1A). The predicted 2D least energy DNA structure was used for follow-up research and analyzed. The interaction between TβR II and S58, made up of nucleotides, was studied by a structure-based approach. The RNA composer online server was first used to construct a closed and open three-dimensional structural model of the nucleic acid aptamer. Then, using the software Amber to perform simple optimization (minimization), we adjusted the corresponding nucleic acid structure, and used Pymol software to convert U in the RNA sequence into T, resulting in the corresponding DNA three-dimensional structure. Finally, the structure of S58 was optimized by using the molecular structure of Gromacs, eliminating the barriers between nucleic acid structures, and extracting nucleic acid structures under the average structure.

### Glaucoma filtration surgery in a rabbit

32 male New Zealand White rabbits (3-5 months, 1.5-2 kg) were subjects of the experiment. All procedures were approved by the Animal Ethics Committee of Chongqing Medical University and met with ARVO ophthalmology and vision studies. Rabbits were randomly administered bilateral drops: Normal group (saline, without surgery) (n = 5), Sham group (saline, surgery) (n = 5), S58 group (4 times/day, 20 nM/drop, postoperative 2 weeks) (Sangon Biotech, Shanghai) (n = 5), the clinical standard mitomycin C (MMC) group (sponge application, 0.02%) (n = 5) (MCE, China), and evaluated at day 0 and 3, 1-week, 2-week, 3-week, and 4-week endpoints. A week before the operation, chloramphenicol was used as an anti-inflammatory for the experimental rabbits. Rabbits were injected intraperitoneally with 3.5 ml/kg 10% chlorine hydrate before surgery, and then 0.5% oxycodone hydrochloride eye fluid were used for local anesthetic. Surgery was performed in bilateral eye of all animals and was based on a previously described technique [51]. The rabbit’s eyes were treated intraoperatively and/or postoperatively with inflammation and scar formation intervention according to the setting group. On postoperative day 14, we sacrificed half of the rabbits and harvested scar tissues from the wounds for histological analysis and western blotting. The remaining rabbits were tested and analyzed for filtering bleb at a predetermined time. At the specified time, the slit lamp (CARL ZEISS, Germany) was used to check the appearance of the filtered bleb and the means of IOP (TonoPen; Medtronic Solan, Jacksonville, FL). The remaining rabbits were tested and analyzed for filtering bleb at a predetermined time.

### Cell culture

Primary human conjunctival fibroblasts (HConFs) (ATCC, USA) were purchased for all cell experiments. The primary HConFs were cultured in DMEM (Hyclone, USA) containing 10% FBS (Gibico, USA), 1% cyan-streptomycin (Biochrom, Berlin, Germany), and 5% CO_2_ at 37°C. The 6^th^ to 12^th^ generation HConFs were used for experiments. Before the cell experiment, the cells were starved in serum-free DMEM for 12 h.

### Histological examinations

On postoperative day 14, 15 experimental rabbits were euthanized, their eyeballs were removed, a small opening was cut on the opposite side of the surgical wound, and eyeballs were immediately placed in a 4% formaldehyde solution, soaked for 48 hours. Tissues were dehydrated, encapsulated, sliced, and used for H&E, Sirius red staining, and Masson’s trichrome staining to evaluate collagen deposition and degree of fibrosis of scar tissues. Sections were also used for immunofluorescence staining; the sections were deparaffinized, rehydrated, and treated with 3% hydrogen peroxide for 10 min at room temperature to eliminate endogenous peroxidase activity. Afterwards, the sections were incubated with 0.4% pepsin (Sangon Biotech, China) for 30 min at 37 °C to retrieve the antigen and subsequently blocked with 1% goat serum albumin for 30 min at room temperature. After incubation with primary antibody against α-smooth muscle actin (α-SMA) (sigma, 1:200), vimentin (Abcam, 1:200), and collagen-1 (sigma, 1:200) overnight at 4 °C, the sections were incubated with an appropriate secondary antibody for 1 h at 37 °C. Images were captured under a light microscope (Olympus Inc., Tokyo, Japan).

### Wound healing assays

To assess the ability of different post-processing (TGF-β 2 (4ng/ml), S58 (20nM)) cells to migrate. HConFs (2 x 10^5^/well) were inoculated in a 6-well plate (Corning, USA) in 5% CO_2_ at 37°C, and the cells were cultured to 80-90% fusion. Making a scratch in the shape of a "#" in each hole with the tip of the sterile 10μl pipette. Subsequently, the HConFs s were washed twice with PBS to remove debris and continued to grow with a serum-free medium for 12h or 24 h. It was then observed and recorded under a microscope (Olympus BX51). And evaluated and analyzed the amount of cell migration at the edge of the scratch.

### RNA interference

The siRNA targeting human TβR II (5’-CCUGUGUCGAAAGCAUGAATT-3’, 5’-UUCAUGCUUUCGACACAGGTT-3’), TβR II negative control siRNA (5’-UUCUCCGAACGUGUCACGUTT-3, 5’-ACGUGACACGUUCGGAGAATT-3), the siRNA targeting human Nrf2 (5’-GCCUGUAAGUCCUGGUCAUTT-3’, 5’-AUGACCAGGACUUACAGGCTT-3’) and Nrf2 negative control siRNA (5’-UUCUCCGAACGUGUCACGUTT-3, 5’-ACGUGACACGUUCGGAGAATT-3) were constructed by GenePharma (Shanghai, China). According to the manufacturer’s instructions, siRNAs were diluted to 20mM with DEPC-treated water (Beyotime, China). We added 10μl siRNA-Nrf2 and 10μl Lipofectamine® 3000 Transfection reagent (Invitrogen, USA) to 500μl DMEM, gently mixed with the pipette head, set aside for 5 minutes, and mixed the same volume at room temperature for 20 minutes, HConFs were washed twice with PBS; we then added the 500μl mixture to each 6-well plate and incubated for 6 h at 37 °C. HConFs were washed with PBS and cultured 72h for follow-up experiments.

### Western blotting analysis

HConFs or tissues were harvested and lysed on ice for 30 minutes with RIPA buffer (Beyotime, China) with a cocktail of protease and phosphatase inhibitors (Thermo Scientific, IL, USA). Total protein was extracted and quantified by BCA kit (Beyotime, China) following the manufacturer’s instructions. Proteins with different molecular weights were separated by sodium dodecyl sulphate-polyacrylamide gel electrophoresis (SDS PAGE) and transferred to polyvinylidene difluoride membranes (Millipore, USA) followed by blocking with western blocking buffer (Beyotime, China). Next, membranes were incubated with primary antibodies against collagen-1 (the ECM deposition level) (Abcam, 1:1000), γ-GCS (Affinity, 1:1000), SOD1 (Abcam, 1:1000), SOD2 (Affinity, 1:1000), Catalase (CAT) (Affinity, 1:1000), p-Nrf2(S40) (CST, 1:1000), Nrf2(CST, 1:1000), smad2/3 (Abcam, 1:1000), p-smad2/3 (Abcam, 1:1000), vimentin (Abcam, 1:1000), α-SMA (Abcam, 1:1000), fibronectin (Abcam, 1:1000), Akt (CST, 1:1000), p-Akt (CST, 1:1000), and Glyceraldehyde-3-phosphatedehydrogenase (GAPDH) (Beyotime, China) overnight at 4°C. At the beginning and the end of incubation with HRP-linked secondary antibody (CST, 1:1000) for 1h on a shaker at room temperature, the blots were washed three times with TBST (Tris Buffered Saline with Tween 20) for 15 min. Band intensity was observed and analyzed relative to the statistical normalization of GADPH, using an enhanced chemiluminescence detection system (Bio-Rad Laboratories) and ImageJ software (Bethesda, MD, USA).

### Real-time PCR

Total RNA was isolated from HConFs using TRIzol®reagent (Cwbiotech, China). Reverse transcription was performed using a Prime Script TMRT kit with cDNA Eraser (#K1622, Thermo Fisher Scientific, Inc.), and RT-qPCR was performed using a SYBR Green qPCR master mix (Takara, Japan). Subsequently, the total reaction volume was 20μl comprised of 2.5 x Real Master Mix/SYBR solution 10μL, PCR forward primer 10μmol/L) 0.5μL, PCR reverse primer (10μmol /L) 0.5μL, cDNA 1μL, ultra-pure water 8μL. Reaction conditions: Total mRNA were 95°C prevaricated 10 min, 95°C denaturation 15s, 62°C annealing 30s, 68°C extension 30s, 50 cycles; and finally, 8 min at 72°C. Relative gene expression was calculated using 2^-ΔΔCt^ method and normalized to GAPDH. (Specific primer sequences are shown in Supplementary Figure 1 and Supplementary Table 1) (Thermo Fisher, USA).

### Cell viability assay

HConFs were inoculated into 96-well plates (4×10^3^cells/well) and pretreated with different concentrations of TGF-β2 for 24h before S58 (20nM) addition for 24h or longer, and then cultured in 100μL of fresh complete medium for 48h. 10μl of CCK-8 reagent (CCK-8, Dojindo, Molecular Technologies) was added per well. After incubation for 1h at 37°C, The absorbance values of each well after incubation was measured by a microplate reader (Multiskan Go Multimode Reader, Thermo Scientific) and statistically analyzed.

### Measurement of ROS

To assess the level of cellular stress, HConFs were inoculated into 6-well plates (4×10^4^ cells-1). HConFs were treated with different treatments for a period of time and washed twice with PBS. The 500μl reaction solution at a concentration of 5x10^-6^m H_2_DCF-DA (Beyotime,1;1000) or MitoSOX Red (5μM, Invitrogen, M36008) for mitochondria-derived ROS evaluation) was incubated in the dark for 20 min at 37 °C. Next, HConFs were trypsinized and resuspended in 150μl of phosphate buffer solution (PBS). Finally, the oxidation of H_2_DCF-DA and MitoSOX Red were detected by flow cytometry and analyzed by Flow-Jo_V10 software.

### Confocal microscopy imaging

HConFs were seeded on 14 mm glass cover-slips (NEST, China) in 24-well plates for 24h. After different pretreatments, the cells were washed twice with cold PBS, and then immediately fixed with 4% paraformaldehyde solution at 4°C overnight. After permeabilization by 0.1% Triton-X100, cells were blocked with 1% goat serum for 1 h at room temperature. The primary antibodies (vimentin/α-SMA/fibronectin) (Abcam, 1:200) (or fluorescence probe (FITC-S58, PE-TβR II)) were incubated overnight at 4°C (or 1h on ice). Next, the slides were washed with PBS and incubated with Alexa Fluor®488- or Alexa Fluor®594-conjugated secondary antibodies (1:500) in the dark for 45 min at 37 °C, followed by DAPI staining for 2 min. Images were captured using a fluorescence microscope (laser scanning confocal microscope, TCS-SP5, Leica) and analyzed.

### Statistical analysis

All data are expressed as mean ± SD. Statistical analysis was applied using one-way ANOVA, and Fisher’s least significant difference test was performed using SPSS21.0 software (SPSS, Inc., Chicago, IL, USA) to compare to determine Statistical significance (P < 0.05).

## Supplementary Material

Supplementary Figures

Supplementary Table 1
