# Supplementary material for: The TβR II-targeted aptamer S58 prevents fibrosis after glaucoma filtration surgery
Source: Aging (Albany NY). 2020 May 23;12(10):8837–57. doi: 10.18632/aging.102997 (PMC7288943; doi:10.18632/aging.102997)
Supplement: Supplementary Figures [file aging-12-102997-s002..pdf]

SUPPLEMENTARY FIGURES

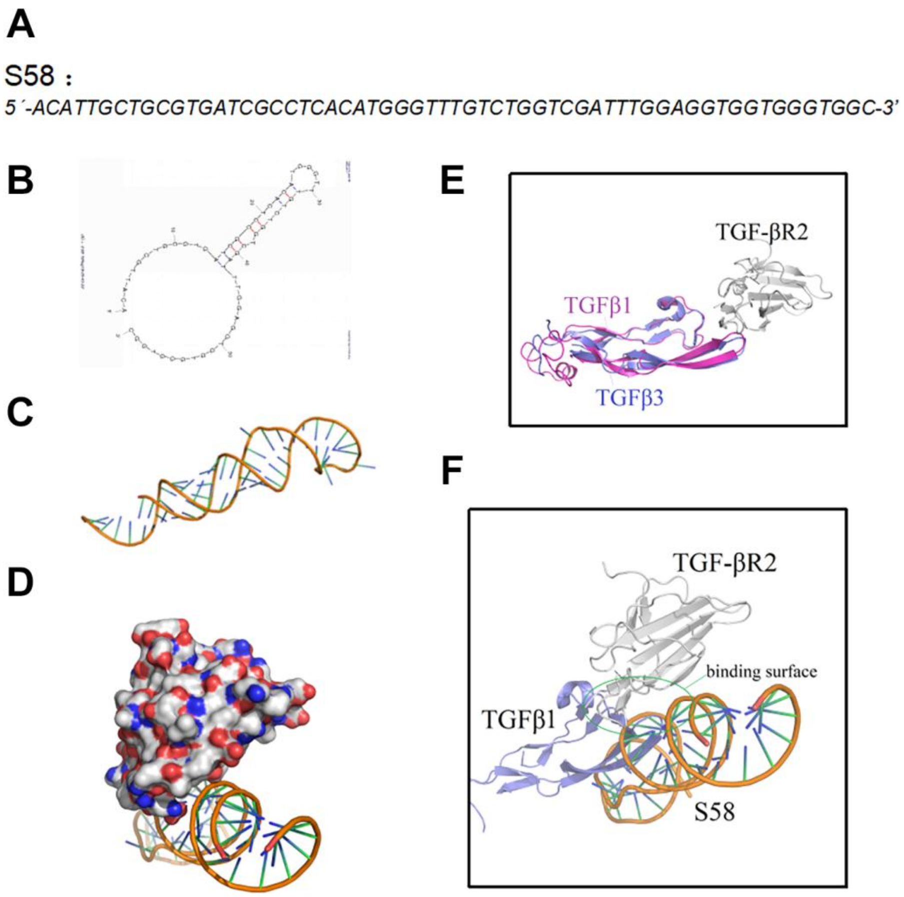

Supplementary Figure 1. (**A**, **B**) Based on the primary structure of the aptamer S58, the DNA mfold program was used to conduct the secondary structure analysis of aptamer S58. (**C**) The most likely binding conformation of aptamer S58 to bind to TRβ II extracellular domain proteins. (**D**, **E**) The nucleic acid adaptor S58 binds to the site of TRβ II, which belongs to the interaction site of TRβ II-TGF-β1/3. (**F**) Conformation superposition of crystalline TβR II, TGF-β1 and S58.

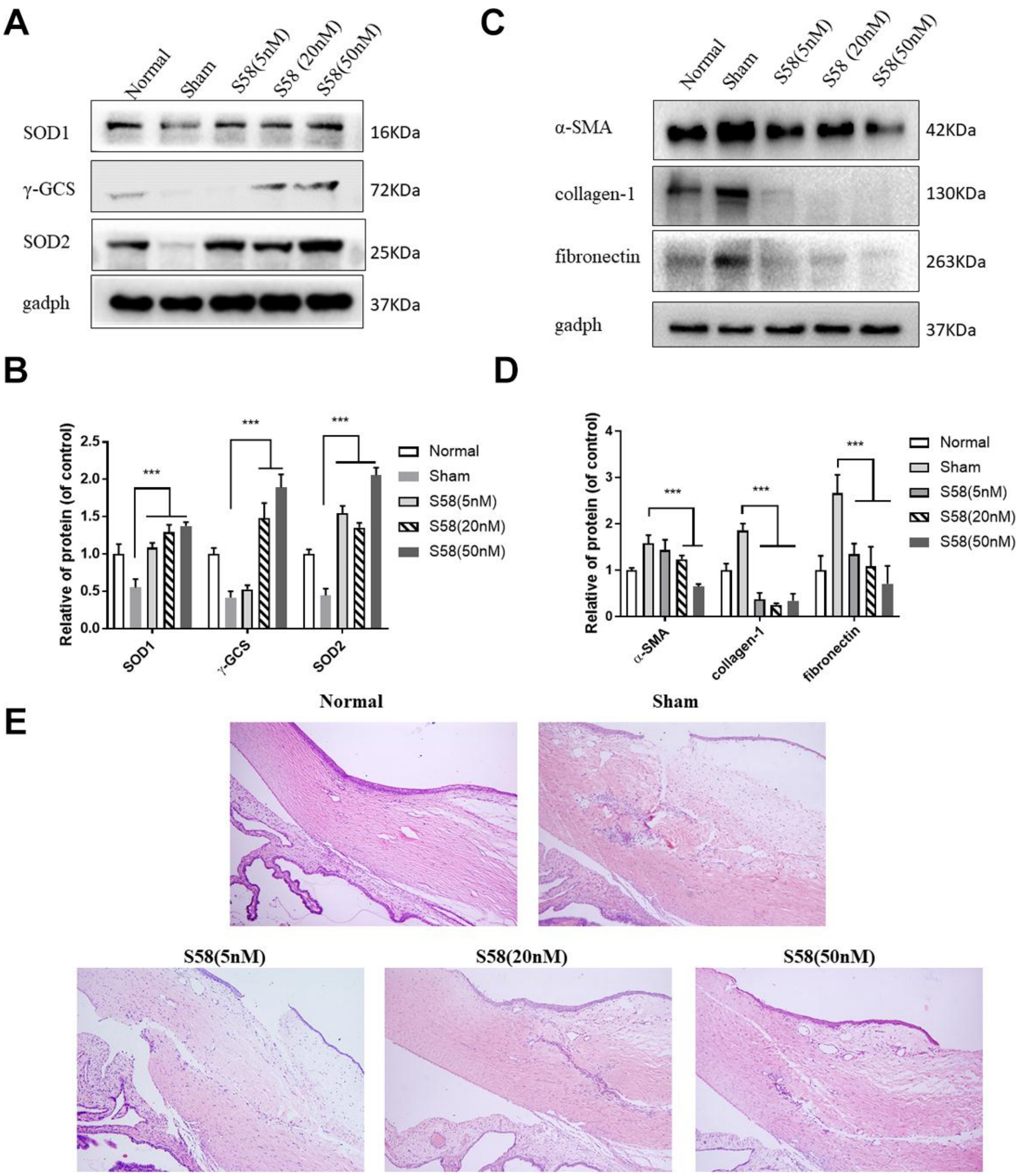

Supplementary Figure 2. Effects of S58 on filtering blebs on postoperative 14 day after GFS. (**A**–**D**) Effects of different doses of S58 (5 nM/drop, 20 nM/drop and 50 nM/drop) on the levels of fibrotic proteins and antioxidants proteins analyzed by western blot and quantification of gray values. (**E**) Representative images of H&E to analyze fibrotic levels.

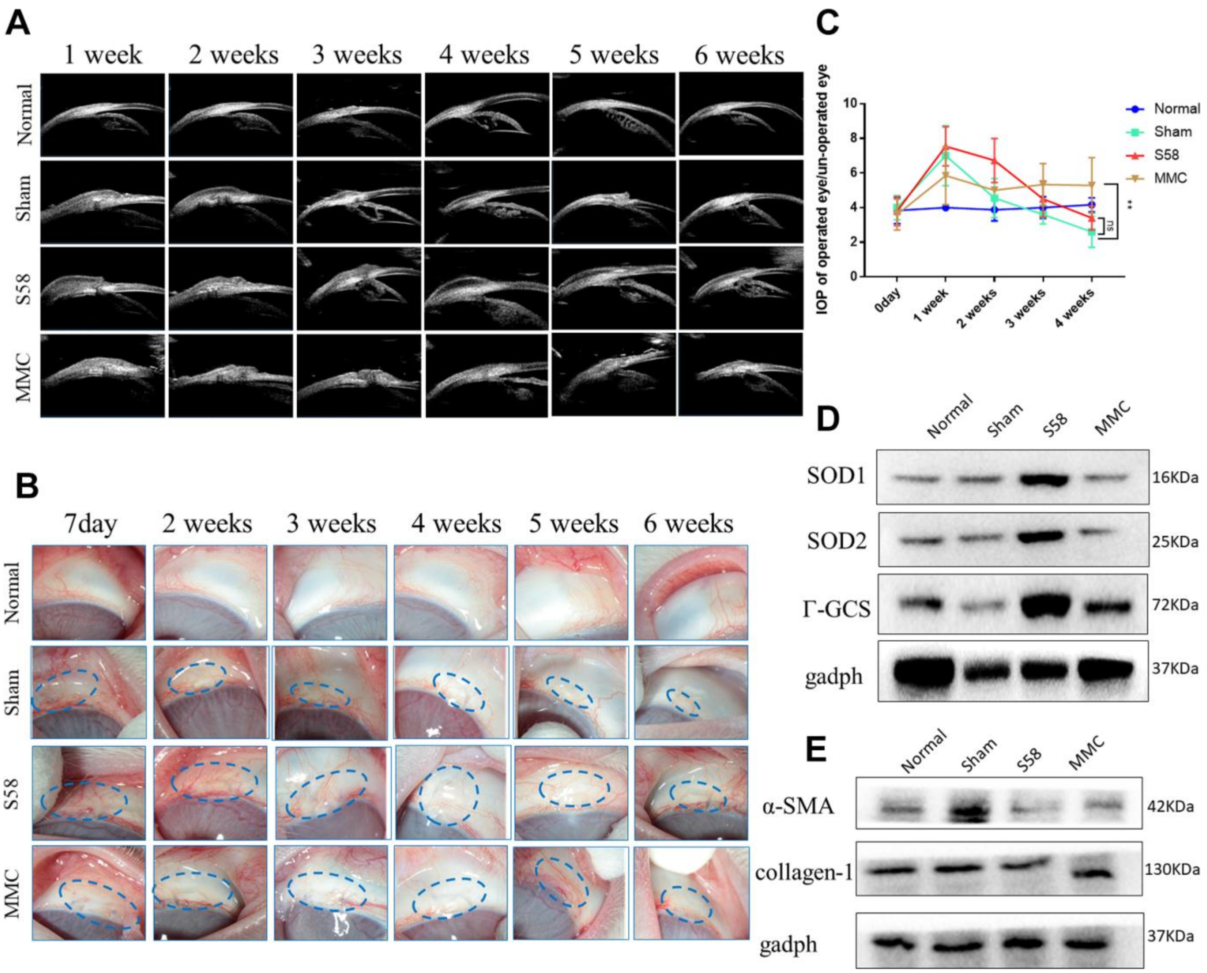

Supplementary Figure 3. Effects of S58 on the filtering blebs in rabbits 6 postoperative weeks after GFS. (**A**, **B**) The appearance and UBM images of filtering blebs in the 4 groups after 6 postoperative weeks. (**C**) IOPs of the operated and non-operated eyes at various time-points. (**D**, **E**) The levels of fibrotic and antioxidants proteins analyzed by western blotting.

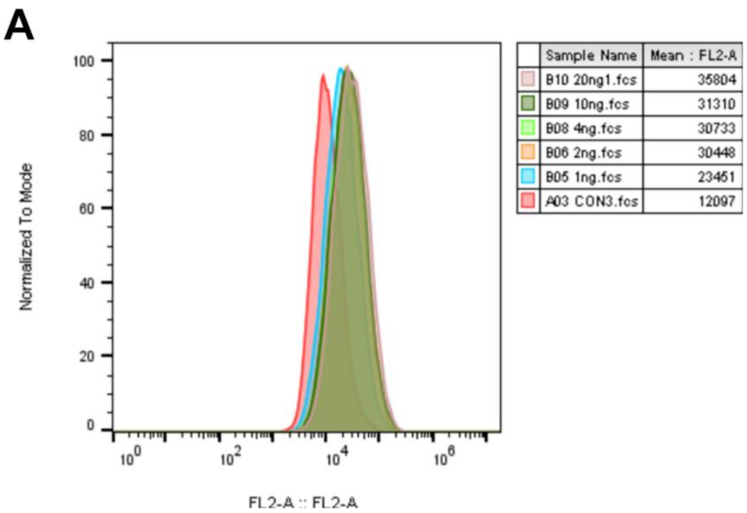

Supplementary Figure 4. (**A**) Intracellular ROS variation at indicated TGF-β2 (4 ng/ml) treatment for 16h.

A

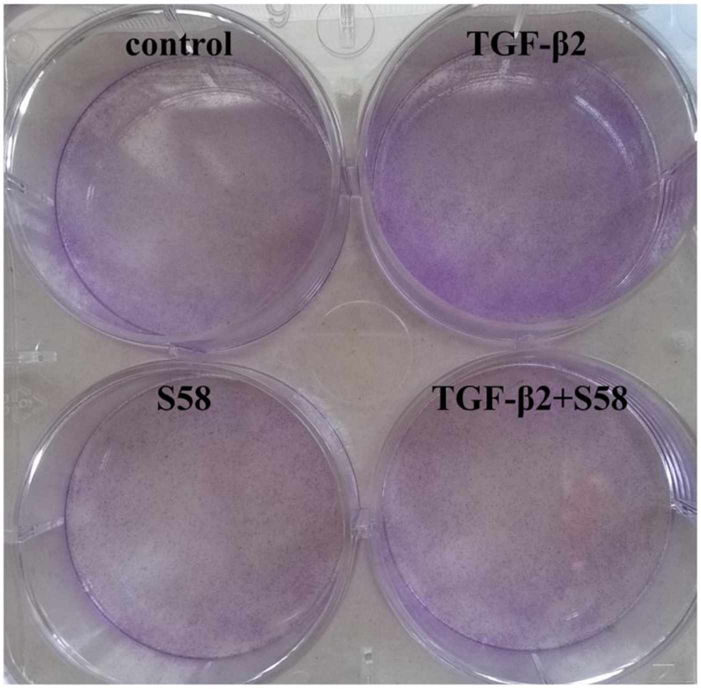

Supplementary Figure 5. (**A**) Representative images of HConFs colonies generated in survival assays, HConFs were treated in the absence or presence of 20 nM S58 for 12 h following TGF-β2 (4 ng/ml) treatment for 3 days (kept with S58).
