# Supplementary material for: The TβR II-targeted aptamer S58 prevents fibrosis after glaucoma filtration surgery
Source: Aging (Albany NY). 2020 May 23;12(10):8837–57. doi: 10.18632/aging.102997 (PMC7288943; doi:10.18632/aging.102997)
Supplement: Supplementary Table 1 [file aging-12-102997-s001..pdf]

SUPPLEMENTARY TABLE

Supplementary Table 1. Primer sequences.

Vimentin/VIM	Forward Primer	GACGCCATCAACACCGAGTT
	Reverse Primer	CTTTGTCGTTGGTTAGCTGGT
FSP-1/S100A4	Forward Primer	ACTTGGACAGCAACAGGGACA
	Reverse Primer	TTCCTGGGCTGCTTATCTGG
CTGF/CCN2	Forward Primer	GAGCCCAAGGACCAAACCG
	Reverse Primer	TGGAGATGCCCATCCCACA
COL3A1 collagen type III alpha 1	Forward Primer	CTTCTCTCCAGCCGAGCTTC
	Reverse Primer	AATGTTTCCTACCGTGCAACC
B-actin/Actb	Forward Primer	CATGTACGTTGCTATCCAGGC
	Reverse Primer	CTCCTTAATGTCACGCACGAT
MMp9	Forward Primer	TACAGGATCATTGGCTACACACC
	Reverse Primer	GGTCACATCGCTCCAGACT
MMp2	Forward Primer	TACAGGATCATTGGCTACACACC
	Reverse Primer	GGTCACATCGCTCCAGACT
TIMP1	Forward Primer	ACCACCTTATACCAGCGTTATGA
	Reverse Primer	GGTGTAGACGAACCGGATGTC
α-SMA/Acta2a	Forward Primer	GATGCTCCCAGGGCTGTTTT
	Reverse Primer	CCTCTTTTGCTCTGTGCTTCGTC
collage1/COL1A1	Forward Primer	GAGGGCCAAGACGAAGACATC
	Reverse Primer	CAGATCACGTCATCGCACAAC
TIMP2	Forward Primer	GCTGCGAGTGCAAGATCAC
	Reverse Primer	TGGTGCCCGTTGATGTTCTTC
fibronectin/FN1	Forward Primer	AGGAAGCCGAGGTTTTAACTG
	Reverse Primer	AGGACGCTCATAAGTGTCACC
N-cadherin/CDH2	Forward Primer	TCAGGCGTCTGTAGAGGCTT
	Reverse Primer	ATGCACATCCTTCGATAAGACTG
